# Therapeutic Notes

**Published:** 1895-02-23

**Authors:** 


### THERAPEUTIC NOTES.

### IODIDE OF POTASSIUM IN HUMAN ACTINOMYCOSIS.

It is now well-known that in veterinary medicine the results of treatment of actinomycosis with potassium iodide have been extremely successful. The disease is much rarer in man than in animals, and few cases have been as yet recorded of successful treatment, but there is no reason to suppose that the parasite will prove more refractory in human beings than in animals. Buzzi and Galli-Valerio (Reform. Med., May 6th, 1893) record a case of actinomycosis affecting the whole of the right side of the face and neck in a man otherwise healthy. It had lasted about four months, and before coming under their observation had been treated in various ways without benefit. As soon as the diagnosis of actinomycosis was made about four hundred and fifty grains of potassium iodide was taken daily by the patient and continued for about two months when the cure was nearly complete. By the end of the third month healing had taken place all over the growth. Improvement began rapidly after commencing the iodide, the swelling and discharge became markedly less, and finally disappeared completely, the skin being freely movable over the underlying parts except at one place. The general health of the patient was excellent throughout, and no unpleasant effects followed the use of such large doses of potassium iodide except a skin eruption. The authors highly recommend the use of large doses of iodide to be kept up until the growths have completely disappeared.

